# The Impact of Sugar Moieties on the Solubility of Naringin-Neohesperidin Co-Amorphous Solid Dispersions

**DOI:** 10.3390/pharmaceutics18081041

**Published:** 2026-08-21

**Authors:** Hua Jiang, Zhong-Kang Yang, Jun Li, Yu-Pin Wang

**Affiliations:** College of Chemistry and Chemical Engineering, Henan University of Science and Technology, Luoyang 471023, China; jianghua@haust.edu.cn (H.J.); 240320251467@stu.haust.edu.cn (Z.-K.Y.); 240320251504@stu.haust.edu.cn (Y.-P.W.)

**Keywords:** naringin, neohesperidin, co-amorphous solid dispersion, sugar moiety, solubility

## Abstract

**Background/Objectives**: Co-amorphous solid dispersions (c-ASD) are a promising strategy for enhancing the dissolution of poorly water-soluble drugs. Naringin (NA) and neohesperidin (NE) are dihydroflavonoid components and are poorly soluble. They can form a c-ASD. Concerning the c-ASD formation mechanism, the intermolecular forces between the non-sugar aglycones of NA and NE have been determined; however, the roles of the sugar chains, which account for nearly 50% of the overall molecular weight of both compounds, are unclear. Therefore, the impact of the sugar moiety on the solubility of NA-NE c-ASD needs to be investigated. **Methods**: The sugar removal products of NA and NE are naringenin-7-O-glucoside, naringenin, hesperetin-7-O-glucoside, and hesperetin. Therefore, in this study, the solubility profiles of NA with hesperetin-7-O-glucoside and hesperetin, and of NE with naringenin-7-O-glucoside and naringenin were assessed by dissolution determination and analyzed by PXRD. **Results**: These results indicated that the rhamnose and glucose moieties on the NE sugar chain and the glucose moiety on the NA sugar chain are vital to the stability and solubility of NE-NA c-ASD. The rhamnose moiety on the sugar chain of NA is removable, without which a stable soluble aggregator may also be constructed, but the soluble aggregator formation ability is weakened. **Conclusions**: The results of this study not only inform the rational selection of co-formers for structurally similar flavanone glycosides but can also help chemists modify the c-ASD structure based on the sugar moiety.

## 1. Introduction

**Figure 1:**
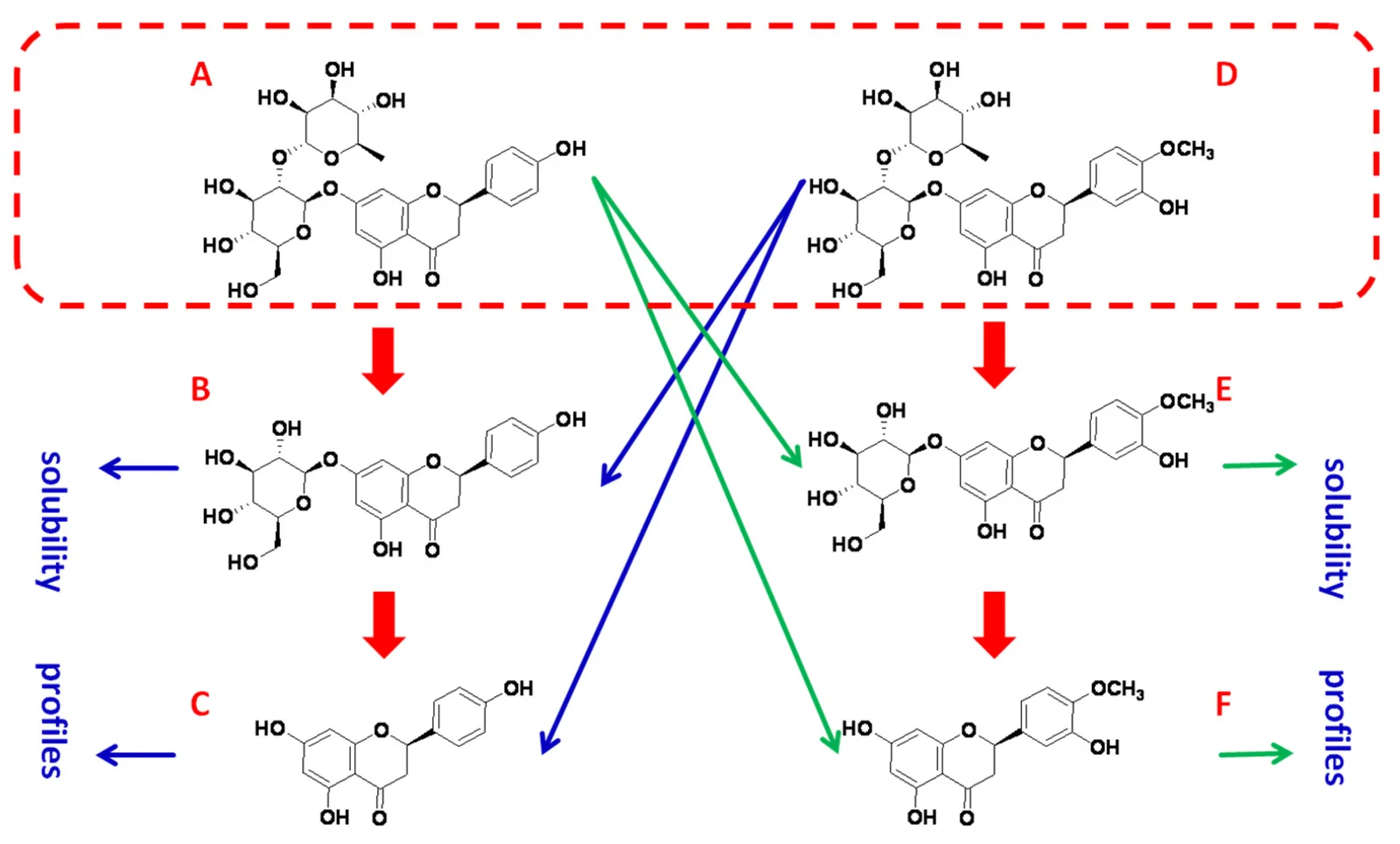
Study framework to identify the core dissolving entity of the bionic co-amorphous solid dispersion system NA-NE; (**A**): naringin, (**B**): prunin, (**C**): naringenin, (**D**): neohesperidin, (**E**): hesperetin-7-O-glucoside, (**F**): hesperetin.

Naringin (NA) and neohesperidin (NE) are flavanone O-glycosides derived from bitter orange (Citrus aurantium). These compounds have similar chemical structures (A and D in Figure 1). The structural differences between them are the substituent groups at the B ring. The B ring of NE is 3′-OH and 4′-OCH_3_, whereas NA has a B ring with an OH located at site 4′.

NE and NA have similar physiological and pharmacological activities, such as anti-inflammatory, anticancer, antiallergic, and antidiabetic activities [1,2,3,4,5,6,7]. NA can effectively inhibit osteoclastogenesis and mature osteoclasts and NE can inhibit the differentiation of adult osteocytes [8,9]. Although NE and NA have positive effects on human health, their use as pharmaceutical agents has been greatly hindered because their water solubilities are quite low. The solubility of NE is 0.31 mg/mL [10], and the water solubility of NA is 4.97 μg/mL [8]. Therefore, a method needs to be developed for enhancing the solubility of these materials.

Co-amorphous solid dispersions (c-ASD) are a promising strategy for enhancing the dissolution of poorly water-soluble drugs, which are single-phase, homogeneous amorphous systems composed of two or more low-molecular-weight components (typically an active pharmaceutical ingredient, API, and one or more small-molecule co-formers) that are intimately mixed at the molecular level. Unlike traditional amorphous solid dispersions (ASDs) that often use polymeric carriers, c-ASDs utilize small-molecule co-formers to stabilize the amorphous state of a poorly water-soluble drug through strong intermolecular interactions, such as hydrogen bonding, ionic interactions, or π-π stacking [11,12]. Representative reported examples are the lacidipine-spironolactone and andrographolide-lysine co-amorphous solid dispersion systems [13,14]. While they offer advantages like enhanced solubility and potentially improved stability, their successful development hinges on the rational selection of a compatible co-former and a thorough understanding of the system’s physical stability [15].

According to the solubility parameter theory, a similar structure implies better miscibility at the molecular level [16], indicating that NE and NA can effectively form a homogeneous single-phase system. Together with our observation of the coexistence of NE and NA at high concentrations in the water-boiled decoction of Fructus Aurantii Immaturus, the dried, unripe fruit of Citrus aurantium L., we developed the bionic NE-NA c-ASD, which reflects the synchronized dissolution of NE and NA [17]. The intramolecular forces in the non-sugars among aglycones are H-bonds between the OH at position 4′ of the NA and the OH at position 3′ of the NE and the pi-pi stacking forces between the B-rings of NE and NA, which play an important role in the formation of the NE-NA co-amorphous pairs.

With enhanced solubility, the NE-NA c-ASD may be used to produce the formulations intended for combination therapies, such as anti-oxidant [18,19] and anti-inflammatory [7,20] pharmacological effects. Moreover, NA may impede the intestinal efflux transporters, eventually enhancing NE intestinal absorption, because NA is an effective P-glycoprotein (P-gp) inhibitor [21,22]. Furthermore, NA has low toxicity, and no adverse effect was observed when administered orally for six consecutive months at a dose of more than 1250 mg/kg/day [23,24,25].

**Figure 2:**
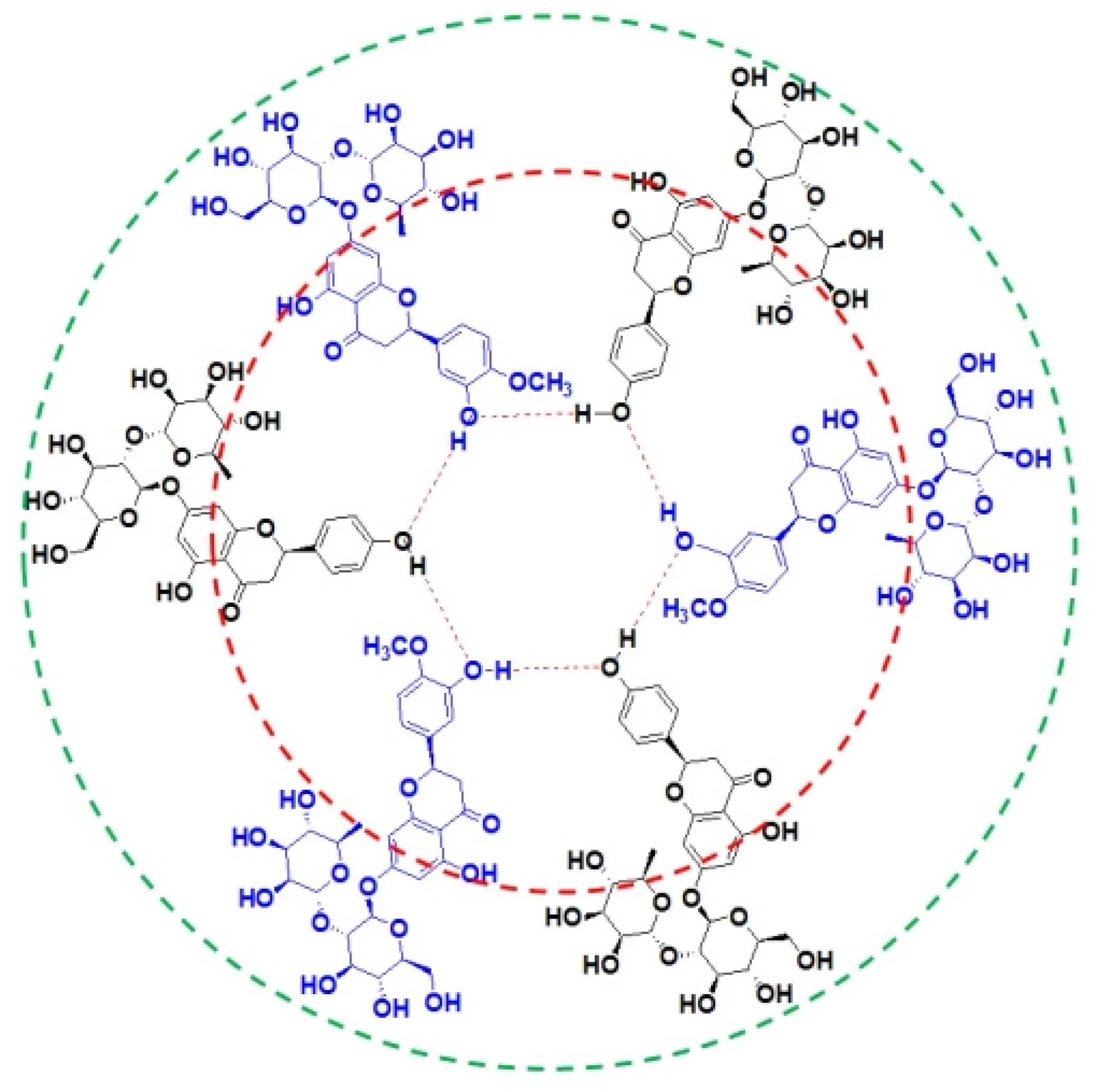
Proposed aggregator structure of NA and NE. The red dotted line indicates the H-bond. The red dashed circle denotes the core of the micelle-like aggregate; the green dashed circle indicates the hydrophilic shell.

Concerning their aggregator structure in water, NA and NE may form a micelle-like structure, in which the hydrophobic aglycones form the core and the sugar chains, whereas the hydrophilic aglycones form the shell, which can form significant hydrogen bonding interactions with water (Figure 2). The above mentioned aggregator structure explains the enhanced water solubility characteristics of NA and NE in the co-amorphous state in water.

NA and NE are both dihydroflavonoid components. These glycosides are composed of an aglycone and a disaccharide sugar chain. Although the intramolecular forces between the aglycones have been described [12], the roles of the sugar chain in the formation of c-ASD are unclear. The sugar chain moiety accounts for nearly 50% of the overall molecular weight of both compounds. Therefore, to gain deeper insights into the mechanism of NA-NE c-ASD formation, we focused on the sugar shell. The aglycone is necessary and responsible for the pharmacological activity. However, glycones are modifiable and can be partly or wholly removed by hydrolysis. Then it is necessary to find the answer to the question that which part of the disaccharide sugar chain in NE is essential or unnecessary to form a c-ASD with NA needs to be determined to improve its water solubility, and vice versa.

By answering this question, this study provided insights into the role of sugars in the formation mechanism of the NA-NE c-ASD system, which can help identify the core dissolving entity. This study also provided essential information on the structure–solubility relationship of the NA-NE micelle-like structure, which can help chemists modify the micelle structure to improve its solubility or find more soluble aggregate structures by removing nonessential sugars.

Many plant-derived bioactive compounds occur as glycosides, which are composed of a sugar chain and an aglycone. Most glycosides suffer from poor water solubility, leading to low bioavailability and limiting their application in food-related and pharmaceutical-related fields. For example, rutin, with a solubility value of 0.125 mg/mL [26,27], possesses many pharmacological activities, such as antioxidant, cytoprotective, vascular, anticarcinogenic, neuroprotective, and cardioprotective effects [26]. Therefore, this study also aimed to provide clues for the development of methods to improve the solubility of such naturally active compounds. Additionally, solubilization theory based on c-ASD could be enriched, especially concerning the rational and effective selection of co-formers for poorly soluble glycosides.

Taken together, a clear delineation of the sugar moiety’s contribution to the solubility of NA-NE co-amorphous solid dispersions (c-ASDs) would advance the understanding of their formation mechanism, support the targeted modification of c-ASD structures, and promote the rational selection of co-formers for enhancing the solubility of glycosides. Accordingly, this work investigated the solubility profiles of NA with the sugar-removed products of NE, including hesperetin-7-O-glucoside (HMG, E in Figure 1) and hesperetin (F in Figure 1), and NE with the sugar-removed products of NA, including naringenin-7-O-glucoside (prunin, B in Figure 1) and naringenin (C in Figure 1), employing dissolution studies and powder X-ray diffraction (PXRD) analysis.

## 2. Materials and Methods

### 2.1. Materials

Crystalline NE (purity ≥ 95%), NA (purity ≥ 95%), HMG (purity ≥ 95%), hesperetin (purity ≥ 98%), prunin (purity ≥ 95%), and naringenin (purity ≥ 98%) were purchased from Yuanyie Biotech Co., Ltd. (Shanghai, China). Methanol (HPLC grade) was purchased from Dikma Tianjin Co., Ltd. (Tianjin, China). Deionized distilled water was used throughout the study.

### 2.2. Sample Preparation

Based on the previous assays [17], the solvent method was adopted for the sample preparation. Briefly, the single prunin, single naringenin, single HMG, single hesperetin samples, and the NE-prunin, NE-naringenin, NA-HMG, and NA-hesperetin samples in weight ratio of 1:1 were weighted and mixed using mortar and pestle for 2 min and then transferred into a round bottom flask containing 200 mL distilled water. The resulting mixture was refluxed at 100 °C for 1 h under nitrogen perfusion. Thereafter, the mixture was put into a freeze dryer (SCIENTZ-30FG, Ningbo Scientz Biotechnology Co., Ltd. Ningbo, China). After thermal equilibration, the shelf temperature was lowered to −30 °C and maintained for 6 h. Subsequently, the system was evacuated to a pressure of 20 torr, and the shelf temperature adjusted to −30 °C and held for 24 h. The shelf temperature was then raised successively to −20 °C (10 h), 0 °C (6 h) and finally 20 °C (2 h). The resulting samples were then weighted and kept sealed at 4 °C until further characterization.

### 2.3. Preparation of pH 7.4 Phosphate Buffer

All reagents were accurately weighed: 48 mM NaCl (2.81 g), 5.4 mM KCl (0.40 g), 2.8 mM Na_2_HPO_4_ (0.40 g), 4.0 mM NaH_2_PO_4_ (0.48 g), and 1.0 g/L D-glucose (1.00 g). The mixture was dissolved in approximately 800 mL of deionized distilled water under continuous magnetic stirring. After complete dissolution, the solution was transferred to a 1 L volumetric flask, and the final volume was adjusted to the calibration mark with additional deionized water. The pH of the resulting solution was verified and adjusted precisely to 7.4 using a calibrated pH meter prior to use.

### 2.4. Dissolution Testing

The powder dissolution profiles over a period of 12 h were conducted using a drug dissolution apparatus (RC809 dissolution tester, Tianjin Tianda Tianfa Co., Ltd., Tianjin, China) by a paddle method with modification [28]. The rotation speed was 50 rpm and 200 mL phosphate buffer pH 7.4 at 37 °C was the dissolution medium. The powder of 1.0 g single prunin, 1.0 g single naringenin, 1.0 g single HMG, 1.0 g single hesperetin samples, and the 2.0 g NE-prunin, 2.0 g NE-naringenin, 2.0 g NA-HMG, and 2.0 g NA-hesperetin samples were dispersed in the dissolution medium and the time was recorded. Samples (2 mL) were withdrawn at different time points (30 min, 1 h, 2 h, 4 h, 6 h, 8 h, 10 h, and 12 h) and immediately replaced with 2 mL of buffer. The samples were filtrated via the 0.45 μm membrane filter, from which 1.0 mL of the filtrate was collected and diluted 10 folds by methanol, then analyzed with HPLC assay. The experiments were conducted in triplicate. The samples used in the dissolution test were freshly prepared and were not subjected to physical stability assessment.

### 2.5. Powder X-Ray Diffraction (PXRD)

Powder X-ray diffraction (HyPix-3000, Rigaku Corporation, Tokyo, Japan) was used to evaluate the solid-state characteristics of the freshly prepared samples. Cu Kα radiation (1.54 °A; 40 kV × 30 mA) was used, and samples were scanned between 5° and 50° 2θ with a dwell time of 0.2 s and a step size of 0.02°.

### 2.6. High-Performance Liquid Chromatography (HPLC) Analysis

HPLC analysis of the samples was performed with a Shimazu Prominence LC-20A System (Shimadzu, Kyoto, Japan) comprising a degasser (DGU-20A5), diode array detection system (SPD-M20A), and a binary gradient pump (LC-20AB). Labsolutions (version 5.97, Shimadzu, Kyoto, Japan) was used to control equipment, obtain and process data, and manage chromatographs. A reversed-phase C18 Wondasil column (4.6 × 250 mm, 5 μm, GL Sciences, Tokyo, Japan) with a matched guard column (Phenomenex, Torrance, CA, USA) were used for analysis. MeOH:H_2_O:H_3_PO_4_ = (27:73:0.03) for NA, NE, HMG and prunin and MeOH:H_2_O:H_3_PO_4_ = (50:50:0.03) for naringenin and hesperetin were used as the mobile phase. The separation conditions were as follows: flow rate 1.0 mL/min, sample injection volume 10 μL, column temperature 30 °C, and UV detector wavelength 280 nm. This HPLC method used in this work was adapted from a previously reported and systematically validated protocol for flavonoid glycoside analysis by Zhang [29]. The method was verified in-house to satisfy standard analytical performance criteria for the quantification of samples.

### 2.7. Statistical Analysis

All measurements were repeated thrice and data are expressed as mean ± SD. Significance testing for intergroup differences was conducted using an independent samples *t*-test, with a *p*-value < 0.05 defined as the threshold for statistical significance. All statistical analyses were performed using SPSS (version 11.0, IBM, Chicago, IL, USA).

## 3. Results and Discussion

### 3.1. Dissolution

#### 3.1.1. Effects of the Sugar-Removed Products of Naringin on the Dissolution Behavior of NE

**Figure 3:**
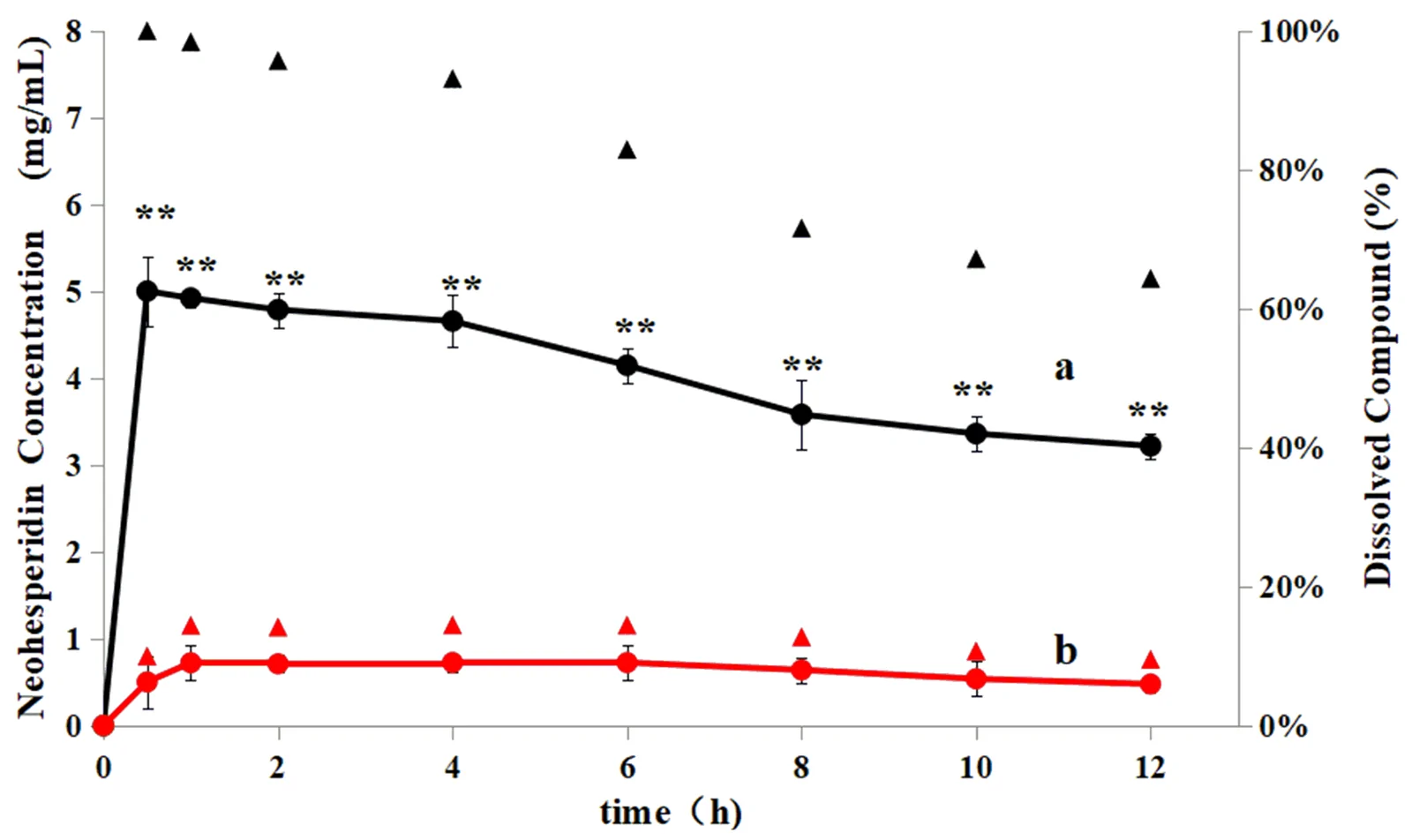
Dissolution profiles of NE from (a) the NE-prunin sample and (b) the NE-naringenin sample. Data are expressed as mean ± SD (n = 3). Statistical significance was analyzed using *t*-test (SPSS 11.0 statistical software). ** *p* < 0.01. The black solid triangles represent the dissolved percentage of NE in (a), while the red solid triangles denote the same in (b).

Two sugar-removed products of NA include naringenin-7-O-glucoside (prunin), the glucose moiety-free product, and naringenin, the sugar chain-removed product or the aglycone (Table 1) [30]. The effects of prunin and naringenin on NE dissolution behavior were illustrated by the NE dissolution curves of the NE-prunin and NE-naringenin samples (Figure 3).

##### Dissolution Behavior of NE in the NE-Prunin Sample

The NE dissolution curve of the NE-prunin sample showed a peak concentration of 5.0 mg/mL after dissolving for 0.5 h, suggesting complete dissolution of NE at this time point (Figure 3(a)). However, the peak concentration of NE did not occur, and the NE concentration decreased to 3.22 mg/mL during the 12 h dissolution period. The NE concentration at the end of the dissolution stage was stable. Consequently, a plausible interpretation is that NE existed in two distinct forms in the NE-prunin sample. One form is modeled as comprising solely NE molecules, which are hypothesized to dissolve easily but possess thermodynamic instability, leading to their eventual precipitation. This model aligns with the observed NE dissolution kinetics: an initial surge in dissolution rate followed by a slow decrease in concentration.

**Figure 4:**
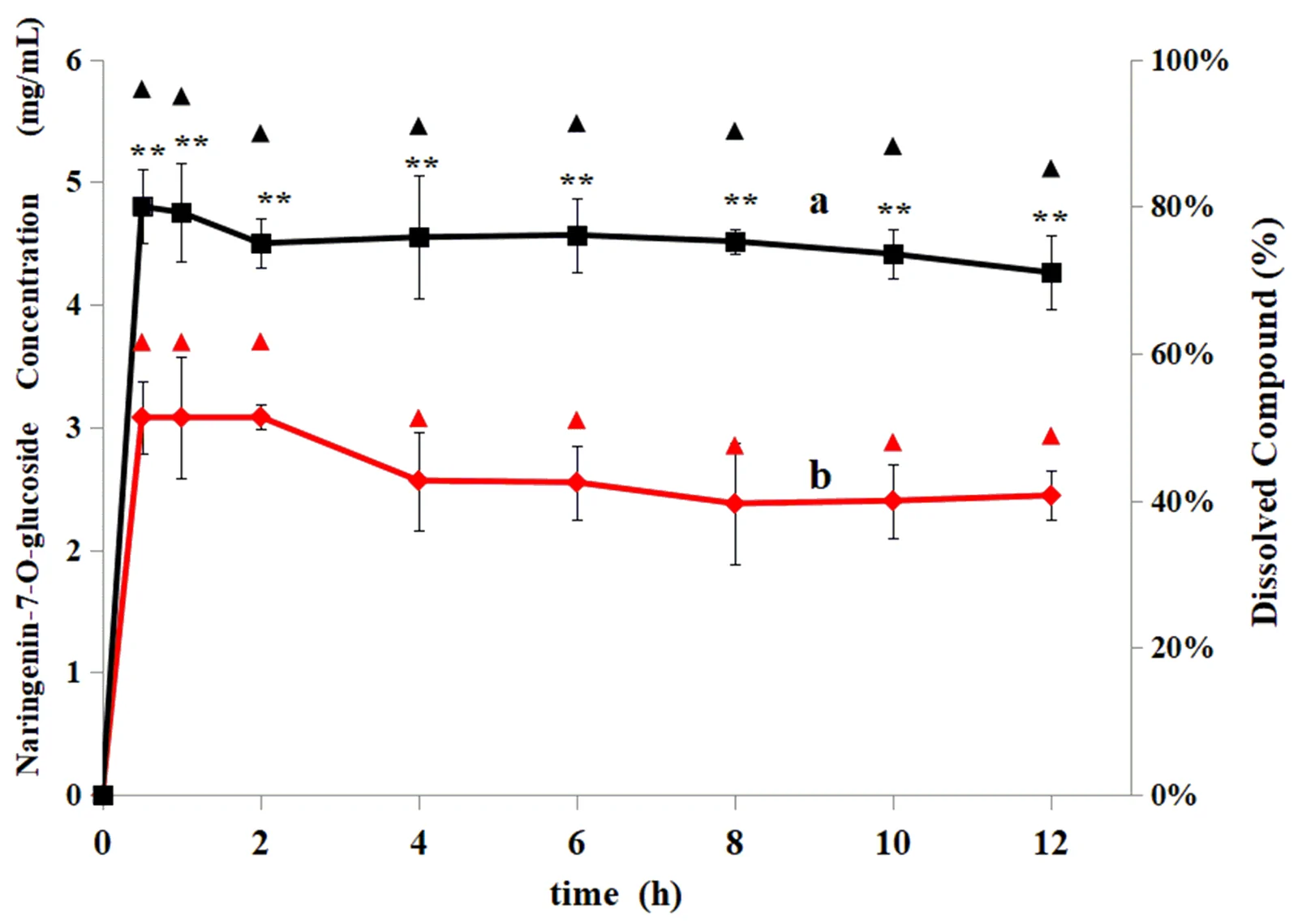
The prunin dissolution profiles of the NE-prunin sample (a) and the single prunin sample (b). Data are expressed as mean ± SD (n = 3). Statistical significance was analyzed using *t*-test (SPSS 11.0 statistical software). ** *p* < 0.01. The black solid triangles represent the dissolved percentage of prunin in (a), while the red solid triangles denote the same in (b).

At the end of the dissolution period, the NE concentration reached a relatively stable value of 3.22 mg/mL. Therefore, the unstable NE accounted for 31% of the total NE weight calculated by the NE sedimentation amount. There was also 69% NE remaining in a dissolved state in the water solution, which indicated the existence of the other forms of NE. This part of NE showed stable dissolving behavior. The prunin in the NE-prunin sample did not show any trend toward sedimentation (Figure 4(a)). Consequently, the data suggest that another fraction of NE may have combined with prunin via intramolecular forces to form soluble and stable aggregators, which could have greatly slowed the precipitation of NE. Analysis of the cumulative dissolution profiles for NE and prunin indicates the probable formation of a water-soluble aggregator with a fixed molar ratio around 1:1.863 (approximately 1:2). From these observations, we infer that prunin—produced by glucose removal from NA—could serve as an effective co-former to NE, potentially enabling the formation of a c-ASD that enhances the water solubility of NE.

As NE and NA at a molar ratio of 1:1 can form a bionic c-ASD [17], the rhamnose moiety at the sugar chain end of NA may not be important for enhancing the solubility of NE. When the rhamnose moiety was removed, the remaining structure of NA also formed a water-soluble and stable aggregator with NE as NE-NA c-ASD. However, the optimized molar ratio changed from 1:1 for NE-NA to about 1:2 for NE-prunin, indicating a weaker ability to form ASD than NA with NE.

Based on the above analysis, the dissolution behavior of NE in the NE-prunin sample may be explained as follows. During sample preparation, the raw materials NE and prunin at a weight ratio of 1:1 (corresponding to a molar ratio of 0.71:1) were dissolved in water and heated at 100 °C. It is inferred that NE may have partitioned into two distinct fractions: one fraction likely reacted with prunin to form an NE-prunin soluble aggregator, while the other fraction is presumed to have transitioned from a crystalline to an amorphous state, which is metastable and tends to precipitate.

However, the inference of a 1:2 molar ratio between NE and prunin as optimal for forming a stable, water-soluble aggregator remains a tentative conclusion, as it is derived from a fixed weight ratio design. This aspect requires careful scrutiny and further experimental validation.

##### Dissolution Behavior of NE in the NE-Naringenin Sample

The NE dissolution curve of the NE-naringenin (the no-sugar product of NA) sample exhibited a low and relatively smooth curve (Figure 3(b)). NE reached its peak concentration of 0.72 mg/mL after 1 h of dissolution, following which no noticeable change in concentration occurred until the 12 h dissolution period. The final NE concentration was 0.48 mg/mL. The concentration of NE decreased by 0.24 mg/mL during the 11 h dissolution period.

The NE dissolution behaviors of the NE-naringenin sample were inferior to that of the single NE sample prepared using the same method as the NE-NA sample [17]. The peak NE concentration of the single NE sample (1.5 mg/mL) appeared at 0.5 h, and its concentration slowly decreased to 1.11 mg/mL after 10 h of dissolution [17]. The concentration at both time points was greater than that of the NE-naringenin sample (*p* < 0.05). The presence of naringenin decreased the water solubility of NE. The high hydrophobicity of naringenin may be the reason. In conclusion, the glucose moiety at site 7 of naringenin is vital for the formation of the NE-NA c-ASD.

Concerning the role of the rhamnose-glucose disaccharide chain of NA in the formation of the NE-NA c-ASD, the rhamnose moiety is not essential, while the glucose moiety is indispensable.

#### 3.1.2. Effects of the Sugar Removal Products of NE on NA Dissolution Behavior

**Figure 5:**
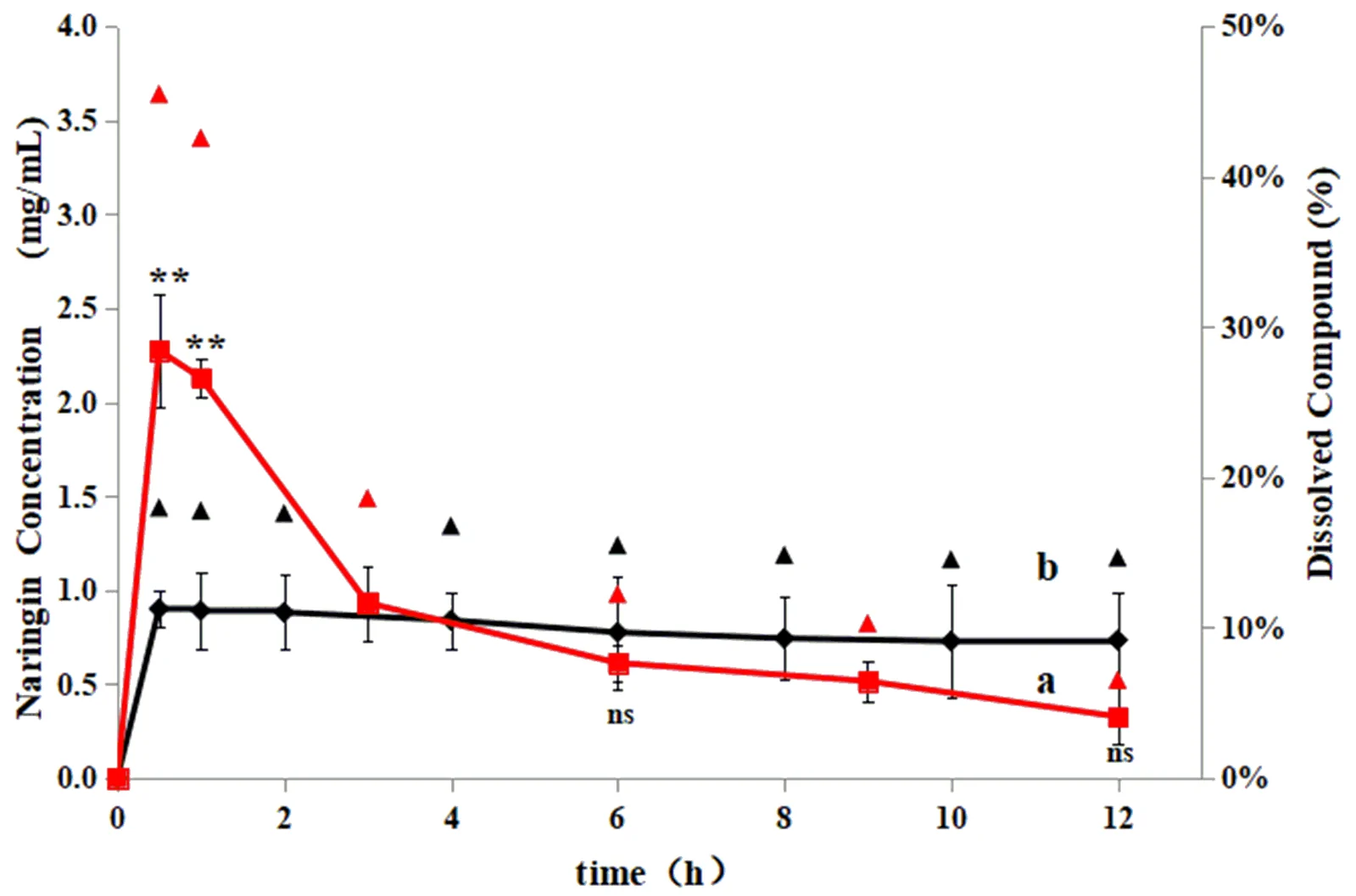
NA dissolution profiles of the NA-HMG (a) and NA-hesperetin samples (b). Data are expressed as mean ± SD (n = 3). Statistical significance was analyzed using *t*-test (SPSS 11.0 statistical software). ** *p* < 0.01, ns: not significant. The red solid triangles represent the dissolved percentage of NA in (a), while the black solid triangles denote the same in (b).

NE has a disaccharide chain composed of glucose and rhamnose at site 7 connected by an ether linkage. There are two sugar removal products, including hesperetin-7-O-glucoside (HMG) [31], the end rhamnose moiety-removed product, and hesperetin, the aglycone or no-sugar product (Table 1). A comparison of the NA dissolution curves of the NA-HMG sample and the NA-hesperetin sample revealed the role of the sugar chain of NE in the formation of the NE-NA c-ASD (Figure 5).

##### Dissolution Behavior of NA in the NA-HMG Sample

When the NA-HMG sample was dissolved in water, the maximum concentration of NA was 2.28 mg/mL at 0.5 h after dissolution, suggesting 46% dissolution of NA at this time point (Figure 5(a)). The NA concentration then decreased quickly to 0.93 mg/mL 3 h after dissolution. The NA concentration of the single NA sample peaked at 0.5 h, with a value of 1.20 mg/mL, followed by a slow decreasing trend, with values of 1.06 mg/mL at 2 h, 0.77 mg/mL at 6 h, and 0.81 mg/mL at 10 h [17]. The NA concentration of the NA-HMG sample in the first three hours was higher than that of the single NA sample (*p* < 0.05). This could be attributed to the potential formation of a soluble aggregator between NA and HMG during sample preparation. However, the intermolecular interactions appeared to be relatively weak and may have been readily disrupted during the aqueous dissolution process, leading to the observed rapid decline in NA concentration. Thus, the rhamnose moiety at the end of the disaccharide chain of the NE is important for the formation of the NE-NA c-ASD. When the rhamnose moiety was removed, the remaining part of the NE, i.e., HMG, could not form a stable and soluble aggregator with NA to increase the solubility of NA.

##### Dissolution Behavior of NA in the NA-Hesperetin Sample

Hesperetin, the aglycone of NE, is hydrophobic. It was produced by removing all sugar moieties from NE [32]. Hesperetin did not increase the solubility of NA, which was manifested by the almost identical dissolution behavior of NA between the NA-hesperetin sample and the single NA sample [17]. The NA dissolution curve of the NA-hesperetin sample revealed its maximum concentration at 0.5 h after dissolution, with a value of 0.90 mg/mL, and in the following 11 h of dissolution, the concentration did not change (Figure 5(b)). No intramolecular force formed between NA and hesperetin. Therefore, the sugar moiety in the structure of NE is important for the formation of stable aggregators among NA and NE.

For the rhamnose-glucose disaccharide chain of NE to play a role in the formation of the NE-NA c-ASD, both the rhamnose moiety and the glucose moiety are important, and the latter is more essential than the former.

To summarize, the rhamnose and glucose moieties on the NE sugar chain and the glucose moiety on the NA sugar chain are important for the formation of NE-NA c-ASD. The absence of any sugar moiety may result in the formation of unstable aggregators, potentially leading to a decrease in concentration or a low solubility in aqueous solution. The rhamnose moiety on the sugar chain of NA is removable. A stable soluble aggregator might also be formed in its absence, but the capacity to form such an aggregator under those conditions could be weakened.

#### 3.1.3. Dissolution Behavior of Prunin

The saturated prunin solubility of a single prunin sample was 2.44 mg/mL, as shown by the stable concentration curve of prunin during the 8–12 h dissolution time interval (Figure 4(b)). The NE-prunin sample achieved a stable prunin concentration of 4.51 mg/mL (Figure 4(a)), indicating its complete dissolution. This value was significantly higher than that of the single prunin sample (*p* < 0.01). NE and prunin appear to form soluble aggregates at a molar ratio of approximately 1:2, which could account for the observed solubility enhancement of prunin in the NE-prunin sample.

#### 3.1.4. Dissolution Behavior of HMG

**Figure 6:**
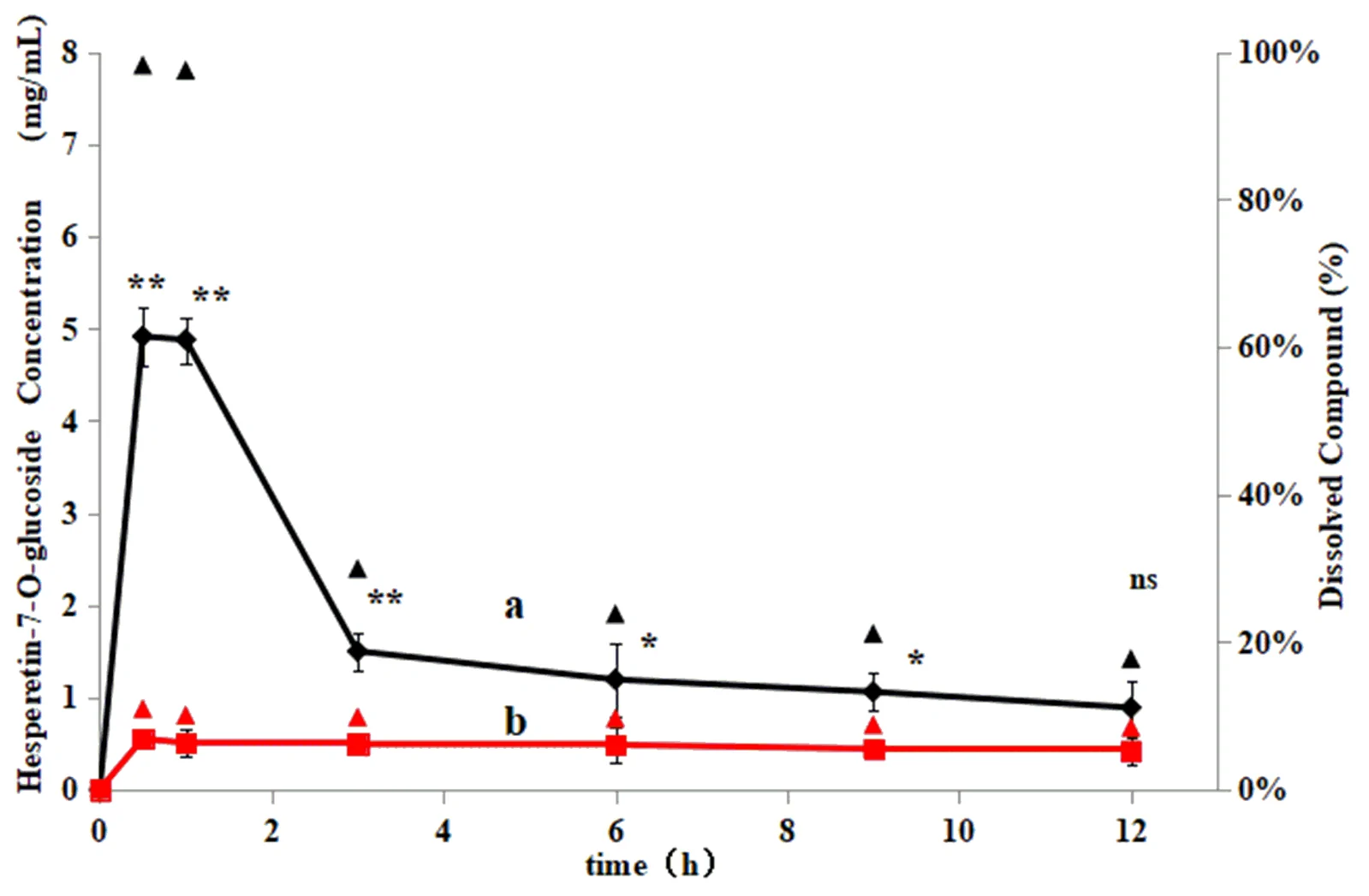
HMG dissolution profiles of the HMG-NA sample (a) and the single HMG sample (b). Data are expressed as mean ± SD (n = 3). Statistical significance was analyzed using *t*-test (SPSS 11.0 statistical software). * *p* < 0.05, ** *p* < 0.01, ns: not significant. The black solid triangles represent the dissolved percentage of HMG in (a), while the red solid triangles denote the same in (b).

The difference in the HMG dissolution curves of the HMG-NA sample and the single HMG sample involved the change in concentration between 0 and 3 h after dissolution. The HMG concentration of the HMG-NA sample reached its peak of 4.92 mg/mL at 0.5 h and quickly decreased to 1.50 mg/mL at 3 h of dissolution (Figure 6(a)). However, the single HMG sample displayed a stable dissolution curve with an HMG concentration of 0.5 mg/mL throughout dissolution (Figure 6(b)). Based on the above results, in the HMG-NA sample, HMG and NA appear to form a soluble aggregator, which may be unstable and prone to disruption during dissolution, potentially leading to the observed significant concentration variation.

#### 3.1.5. Dissolution Behavior of a Single HMG Sample and a Single Prunin Sample

We determined the differences in dissolution behavior between a single HMG sample and a single prunin sample. The structural difference between HMG and prunin is small and lies in the B ring. HMG has a B ring with an OH at 3′ and an OCH_3_ at 4′; however, prunin has a B ring with an OH at 4′. The remaining parts of both compounds are identical. Although the difference between HMG and prunin is small, the difference in dissolution behavior between them is large. As shown in Figure 4(b), prunin reached its peak concentration of 3.08 mg/mL at 0.5 h after dissolution, and this concentration remained high until 2 h and then slightly decreased to 2.56 mg/mL at 4 h. Then, the prunin concentration remained at 2.4 mg/mL until the end of the dissolution period. In contrast, HMG showed a relatively low concentration of 0.49 mg/mL throughout the dissolution period (Figure 6(b)). From the single-molecule perspective, the dissolution difference occurred due to the structural difference. Although the OCH_3_ at the B ring is a hydrophobic group used for the differentiation between HMG and prunin, it is too small compared to the entire molecule. Therefore, OCH_3_ cannot sufficiently or properly explain the difference.

We explained the different dissolution behaviors of HMG and prunin by focusing on multimolecular aggregation rather than the single-molecule structure. During the initial boiling step in water, the material may have undergone self-aggregation or potentially formed aggregators with other compounds present. The aggregator had properties different from those of its original crystal material. A detailed discussion is provided in Section 3.3.

#### 3.1.6. Dissolution Behavior of Naringenin and Hesperetin

**Figure 7:**
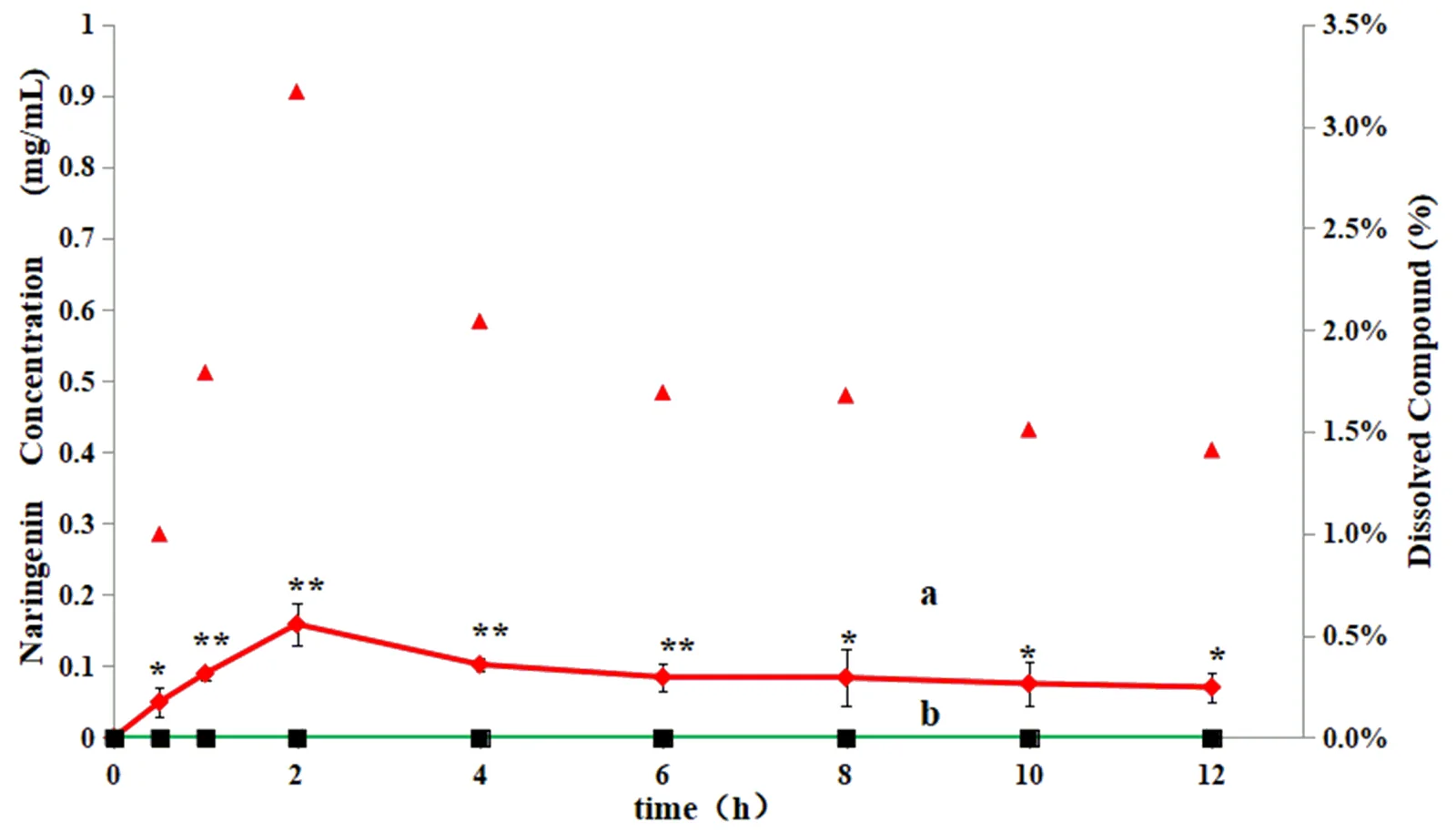
Naringenin dissolution profiles of the NE-naringenin sample (a) and the naringenin sample (b). Data are expressed as mean ± SD (n = 3). Statistical significance was analyzed using *t*-test (SPSS 11.0 statistical software). * *p* < 0.05, ** *p* < 0.01. The red solid triangles represent the dissolved percentage of HMG in (a).

Both naringenin and hesperetin in the single-material samples displayed a linear dissolution curve when the concentration was 0 mg/mL, indicating that they are insoluble in water (Figure S2(b) and Figure 7(b)). The naringenin dissolution curve of the naringenin-NE sample exhibited a peak concentration of 0.16 mg/mL after 2 h of dissolution and then decreased to 0.07 mg/mL after 6 h of dissolution, after which it remained at 0.07 mg/mL until the end of dissolution (Figure 7(a)). The observation of a higher peak naringenin concentration in the naringenin-NE sample, compared to the naringenin-only sample (*p* < 0.01), suggests the possible formation of more soluble aggregates between naringenin and NE. However, the formation of naringenin-NE aggregates appears to be challenging, and the aggregates that do form may be relatively unstable. The hesperetin dissolution curve of the NA-hesperetin sample was identical to that of the single hesperetin sample, indicating that no aggregation occurred between the NA-hesperetin samples.

### 3.2. PXRD

**Figure 8:**
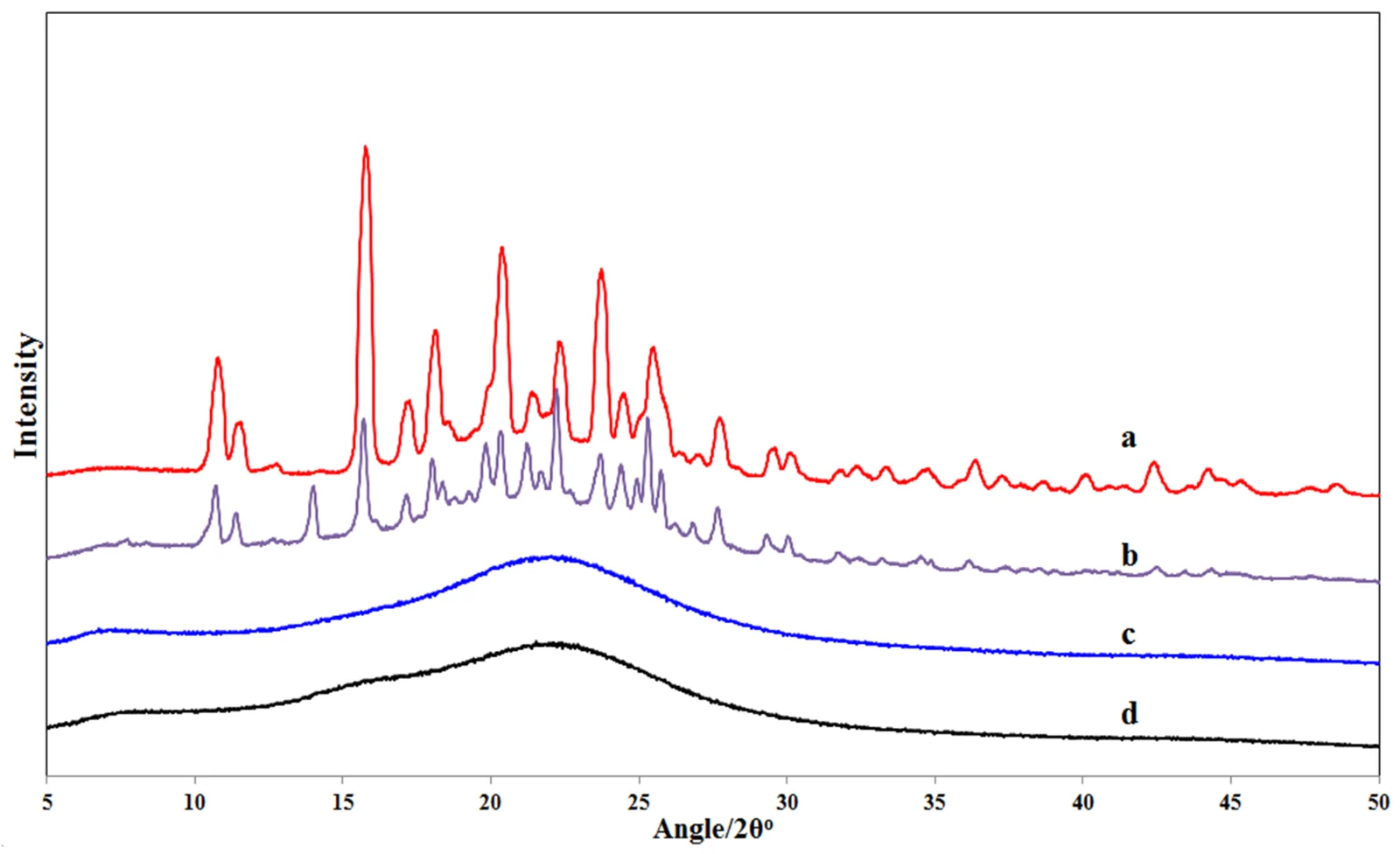
PXRD patterns of naringenin (a), NE-naringenin (b), prunin (c), and NE-prunin (d).

The NA-NE c-ASD is in a single-phase amorphous form. The PXRD technique was used to confirm the degree of crystallinity present in the samples by the diffraction peak profile [33]. To explore the effect of the sugar chain of NA on the amorphous state of NA–NE c-ASD, the PXRD patterns of the NE-prunin sample and the NE-naringenin sample were compared. Prunin, a rhamnose moiety that removes the product of NA, can form an amorphous solid dispersion with NE, as shown by the PXRD profile, with no diffraction peak (Figure 8(d)). Therefore, in the NE-prunin sample, both compounds existed in an amorphous state. Based on the dissolution data, prunin appears to form a stable soluble aggregator with NE in the NE-prunin sample, at an approximate molar ratio of 2:1. This indicated that prunin had a relatively weaker ability to form an amorphous solid dispersion with NE than NA, which was present at a molar ratio of 1:1. Therefore, removing the rhamnose moiety on the end sugar chain of NA did not destroy the co-amorphous feature of the NE-NA cASD but increased the mole percentage of prunin, the NA-related co-former, in the amorphous form with NE. This indicated a relatively weaker ability to form an amorphous solid with NE than with NA.

In the NE-prunin sample, the weight ratio was 1:1, which was equal to the molar ratio of 0.71:1. NE and prunin appear to form aggregators at a molar ratio of approximately 0.5:1. Consequently, it is estimated that an excess of about 0.21 molar portion of NE in the sample may not have participated in the formation of the NE-prunin aggregator. The excess NE was also in an amorphous state, which was indicated by the presence of an amorphous halo in the PXRD diffractogram of the sample (Figure 8(d)). However, the single NE sample presented relatively weaker peaks in its diffractogram than its crystalline form [12]. The excess NE in the NE-prunin sample quickly dissolved and then precipitated out of the solvent, which was also different from the single NE sample dissolving behaviors with a low concentration, similar to NE crystalline form. Therefore, in the NE-prunin sample, NE and prunin were assumed to form compound aggregators at a molar ratio of 1:2. and when NE was in excess, the NE formed a soluble but unstable amorphous solid, which was different from its single sample. Then, the excess NE in a sample cannot be regarded as a separate part of the sample.

The NE-naringenin sample showed peaks in the diffractogram, and the positions of all peaks in the diffractograms (Figure 8(b)) were similar to those of the single naringenin sample (Figure 8(a)) and single neohesperidin sample [17], suggesting that NE and naringenin were in their crystalline forms. However, the dissolution results showed that in the NE-naringenin sample, NE and naringenin presented low peak concentrations of 0.72 mg/mL and 0.15 mg/mL, respectively, 1 h after dissolution, indicating that the formation of NE-naringenin-soluble aggregates was difficult. Therefore, in the NE-naringenin sample, most of the NE and naringenin molecules were in their crystalline forms, and the remaining minority was in an unstable aggregator form. Naringenin is the sugar chain-removed product of NA. Therefore, the sugar chain on NA is required for the formation of NE-NA c-ASD. Compared to the prunin in the NE-prunin sample, the glucose moiety on the sugar chain on NA is particularly important for the formation of NE-NA c-ASD.

The NE-prunin sample and the single prunin sample exhibited an amorphous halo in their PXRD diffractograms (Figure 8(c,d)). Therefore, prunin is prone to transforming from its crystalline form to its amorphous form. Moreover, its ability to form an amorphous state is so strong that it can form a co-amorphous form with NE, which cannot be easily converted from its crystalline form to an amorphous form [17].

The single naringenin sample presented diffractogram peaks in the PXRD pattern shown in Figure 8(a), and its dissolution results revealed that it was insoluble (Figure 7(b)), which indicated that naringenin cannot be easily converted to an amorphous form. NA can be converted to an amorphous form [17]; thus, the sugar chain is important for the formation of the amorphous form of NA.

**Figure 9:**
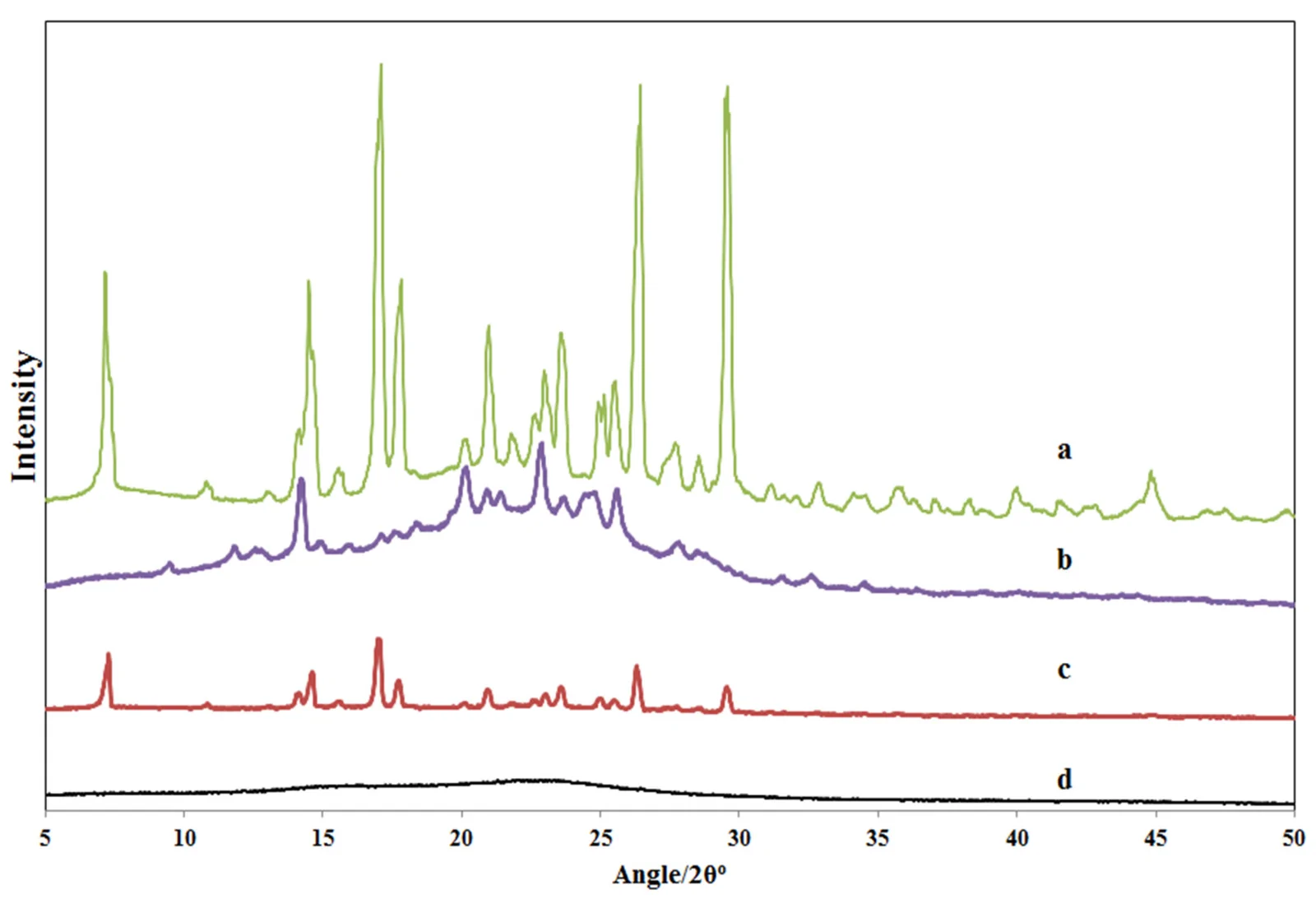
PXRD patterns for hesperetin (a), HMG (b), NA-hesperetin (c), and NA-HMG (d).

The NA-HMG sample was converted to an amorphous form, as indicated by the amorphous halo in the diffractogram (Figure 9(d)). The dissolution results of the NA-HMG sample indicated that NA and HMG reached peak concentrations of 2.28 mg/mL (Figure 5(a)) and 4.92 mg/mL (Figure 6(a)), respectively, at 0.5 h after dissolution. The peak concentrations were higher than those of the single NA and HMG samples (*p* < 0.01), with values of 1.20 mg/mL and 0.55 mg/mL, respectively. Thus, NA and HMG appeared to form an amorphous aggregate, improving their solubility at an approximate molar ratio of 1:2.7. The sample had excess NA, which did not form aggregates with HMG. The PXRD profile indicated that the excess NA was also in an amorphous state. Moreover, the NA concentration decreased after it reached a peak value, indicating that the formed NA-HMG aggregator was unstable. At the end of dissolution, the concentrations of NA and HMG were 0.33 mg/mL (Figure 5(a)) and 0.89 mg/mL (Figure 6(a)), respectively, which were similar to the concentrations of their individual samples. Therefore, HMG may form an unstable amorphous aggregator with NA, which was different from the stable amorphous aggregator formed by NE and prunin. Thus, the rhamnose moiety on the sugar chain of NE is more important than its counterpart on NA for the formation of NE-NA c-ASD.

The NA-hesperetin sample showed peaks in the diffractogram (Figure 9(c)), and the positions of all peaks in the diffractograms were similar to those of the hesperetin sample (Figure 9(a)), suggesting that the hesperetin in the NA-hesperetin sample was in its crystalline form and the NA was in its amorphous form. However, the dissolution results of NA indicated that its amorphous form was unstable and quickly precipitated out of the solution because the dissolution curve of NA (Figure 5(b)) was similar to that of its single sample [17]. Moreover, hesperetin in the NA-hesperetin sample also resulted in a dissolution curve approaching 0 mg/mL throughout the dissolution period (Figure S2(a)), confirming that NA did not form aggregates with hesperetin. Without the glucose moiety on NE, it cannot form a stable amorphous aggregate with NA. The ability of the glucose moiety on the sugar chain of NE to form stable amorphous aggregates with NA is important.

While the PXRD patterns confirm the amorphous nature of the prepared samples, a notable limitation of the current study is the absence of complementary thermal analysis by differential scanning calorimetry (DSC). This prevents direct confirmation of the single-phase co-amorphous state and determination of the glass transition temperature (Tg), which are key parameters for fully characterizing such systems and predicting their physical stability. Future studies will incorporate DSC to address this gap.

### 3.3. Aggregator Model Interpretation

#### 3.3.1. Solubility of the NE-Prunin Sample

Based on the observed dissolution profiles and PXRD patterns, we here propose hypothetical aggregate models for different samples, which aim to illustrate the potential role of the sugar moiety in the formation of NA-NE c-ASD.

**Figure 10:**
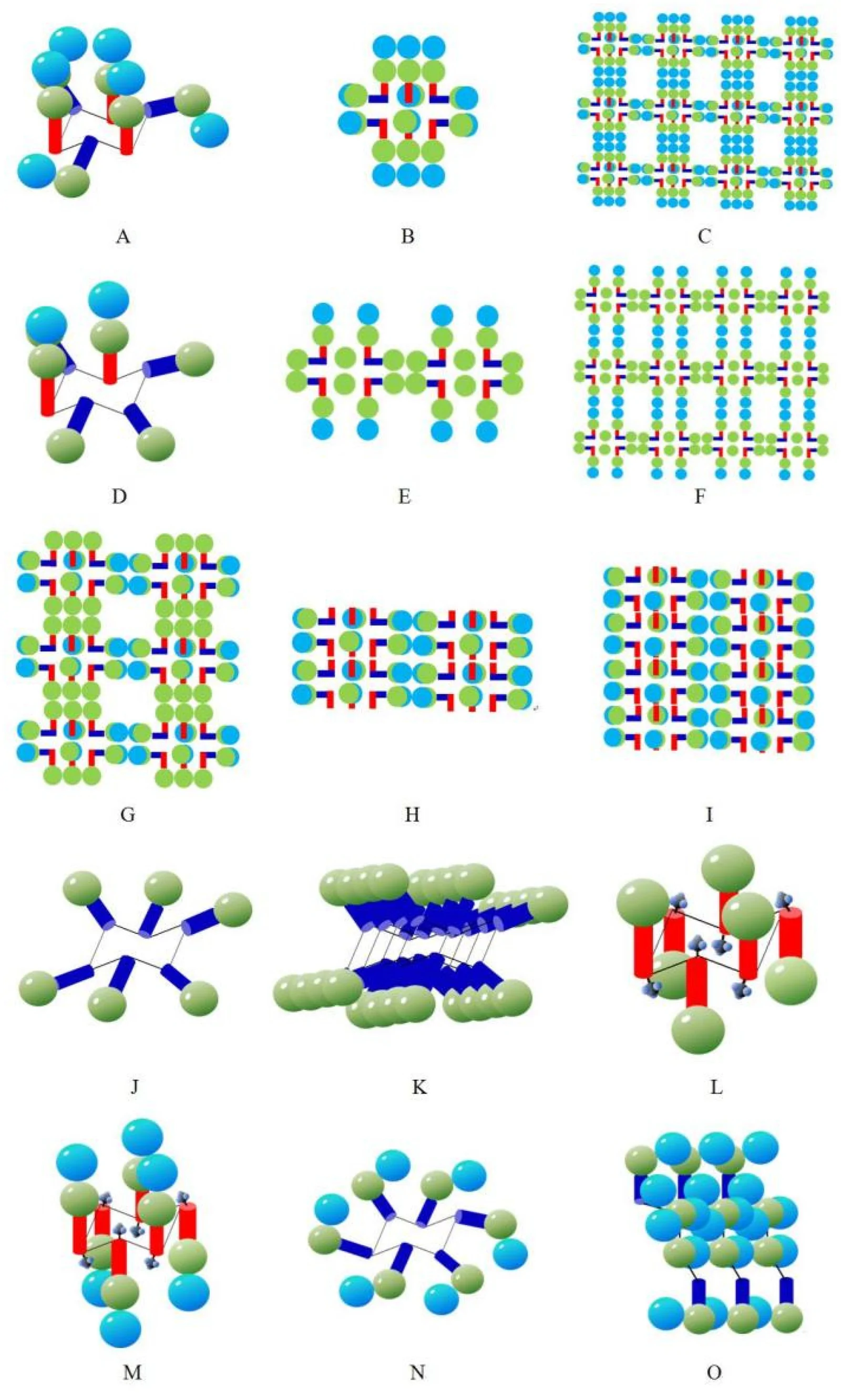
Proposed hypothetical aggregate models. The simplified aggregator model of NE- NA c-ASD (**A**), the dimer structure of NE- NA c-ASD (**B**), the lamellar structure of NE-NA c-ASD (**C**), the simplified aggregator model of NE-prunin c-ASD at a molar ratio of 1:2 (**D**), the dimer structure of NE-prunin c-ASD at a molar ratio of 1:2 (**E**), the lamellar structure of NE-prunin c-ASD at a molar ratio of 1:2 (**F**), the proposed lamellar structure of HMG-NA (**G**), the proposed dimer of hesperetin-NA (**H**), the proposed lamellar structure of hesperetin-NA (**I**), the simplified aggregator model of prunin (**J**), the proposed structure of the prunin-based aggregator aggregator (**K**), the simplified aggregator model of HMG (**L**), the simplified aggregator model of NE (**M**), the simplified aggregator model of NA (**N**), and the proposed lamellar structure of the poly NA aggregator (**O**). The blue cylinder denotes naringenin, the aglycone of NA; the red cylinder denotes hesperetin, the aglycone of NE; the green ball denotes the glucose moiety; the blue ball denotes the rhamnose moiety; the black line denotes the H-bond.

As the foundational starting point for our hypothetical model development, we first focused our exploratory efforts on the NE-NA c-ASD system. The intramolecular forces were H-bonds between the OH at position 4′ of NA and the OH at position 3′ of NE and pi-pi stacking forces between the B-rings of NE and NA in the hydrophobic core [17]. To tentatively illustrate the plausible spatial arrangement of NE-NA molecules in the c-ASD form, a hypothetical molecular aggregate model consistent with our experimental observations is proposed, as depicted in Figure 2. In this model, H-bonds between three NE and three NA molecules at a molar ratio of 1:1 formed a stable six-member ring in the chair conformation as cyclohexane, and the pi-pi stacking was offset face-to-face. Figure S1A visualizes a preliminary hypothetical overview of the NE-NA aggregator, a tentative construct we propose to comprise three NE and three NA molecules in a plausible, experiment-aligned spatial arrangement. The simplified aggregator model is illustrated in Figure 10A. The hydrophobic core consisted of six aglycones, and the hydrophilic outer shell consisted of the sugars.

Consistent with the observed experimental trends, we tentatively propose that as more of these hypothetical NE-NA aggregators build up in aqueous environments, two individual aggregators may first combine to form a putative dimer (Figure 10B), driven by the mutual attraction of their hydrophobic cores to reduce unfavorable hydrophobic interactions [34]. This initial dimerization step could subsequently lead to the emergence of an extended, complex lamellar assembly that exhibits micelle-like characteristics (Figure 10C).

Building on the simplified hypothetical aggregator framework established for the NE-NA c-ASD system, we tentatively propose corresponding non-definitive models for the simplified aggregator and dimer structure of the 1:2 molar ratio NE-prunin c-ASD, as visualized in Figure S1B and Figure 10D, respectively. As suggested by the hypothetical lamellar structure in Figure 10F, the removal of the rhamnose moiety from the sugar chain on NA in our model may not result in a reduced horizontal distance between the two NE-prunin dimers (Figure 10E). This plausible trend can be rationalized by the proposed arrangement in Figure 10A, where the glucose and rhamnose moieties on the disaccharide chain of NA are oriented in a nearly vertical, rather than horizontal, direction. Within our proposed hypothetical model framework, the tentative horizontal spacing between adjacent dimers in the NE-prunin c-ASD assembly appears nearly comparable to the corresponding value in the NE-NA c-ASD system. This observation from the schematic model provides preliminary, speculative support for the idea that NE and prunin are capable of forming a stable aggregator at a 1:2 molar ratio.

#### 3.3.2. Solubility of the HMG-NA and Hesperetin-NA Samples

To establish a working, non-conclusive framework for exploring the mechanistic role of the NE sugar chain in NE-NA c-ASD generation, we advance hypothetical molecular aggregate models that are constrained to align with all collected experimental observations.

As suggested by the proposed hypothetical lamellar structure for HMG-NA in Figure 10G, the tentative vertical spacing between adjacent dimers in this model appears to decrease following the removal of the rhamnose moiety from the NE unit. Using the putative molecular distance in the hypothetical NE-NA c-ASD assembly (Figure 10C) as a baseline stable reference, our proposed non-definitive model suggests that the HMG-NA aggregator may carry a significantly stronger hydrophobic effect, a factor that could plausibly compromise the overall stability of the system. Following this speculative line of reasoning, when the HMG-NA aggregator is exposed to water, HMG and NA may gradually precipitate out of the solution once their concentrations exceed the peak solubility thresholds, a trend that would imply the breakdown of the HMG-NA aggregator framework.

Similarly, hesperetin and NA may not form stable aggregators in our hypothetical model, as the shorter vertical distance between dimers in the proposed hesperetin-NA dimer (Figure 10H,I) could generate a greater hydrophobic effect when the hydrophobic hesperetin units come closer.

#### 3.3.3. Solubility of the Prunin and HMG Samples

In our non-definitive hypothetical structural framework, we propose that six prunin molecules may assemble into a homogeneous hexamer, with the stabilization provided by intermolecular H-bonding and π–π stacking forces (Figure 10J). The H-bonds established by the B ring hydroxyl groups between neighboring prunin units could potentially generate a six-membered ring adopting a stable chair conformation. In this speculative arrangement, each prunin molecule is situated roughly along the equatorial region of the hypothetical six-membered ring. As more of these hexameric units align in a parallel fashion, they may further aggregate into a hollow rod-shaped assembly (Figure 10K). This tentative aggregator is hypothesized to possess a hydrophobic interior formed by the aglycone naringenin units, while the hydrophilic glucose moieties constitute the outer shell, thereby imparting a hydrophilic nature to the overall structure, which would offer a reasonable, model-based rationale for prunin’s high aqueous solubility.

The low HMG concentration of the single HMG sample indicated the impossible formation of the hydrophilic HMG aggregator. On the contrary, prunin with a similar structure as HMG formed a soluble aggregator. This distinction stems from a structural variation: the presence of an OCH_3_ group on the B ring. Additionally, both prunin and HMG possess an OH group on the B ring, but the specific positions differ: the hydroxyl group is located at the 4′ position in prunin, whereas it is at the 3′ position in HMG. In our hypothetical model, prunin’s H-bonds form a six-membered ring in a chair conformation, a potentially energy-favorable state. For HMG, the 3′-OH position (Figure 10L) may force the six molecules to align parallel to a vertical axis through the ring center, with alternating up/down orientations. The six OCH_3_ groups, at the aglycone ends, are distributed three above and three below the hexamer equator. This crowded arrangement could lead to high internal energy, potentially preventing stable HMG hexamer formation and explaining its low water solubility.

#### 3.3.4. Solubility of Aglycones, Monoglycosides, and Diglycosides of NE and NA

A comparison of the dissolution curves of NE and its two sugar removal products, HMG and hesperetin, revealed that the water solubility sequence from high to low was NE [17], HMG (Figure 6(b)), and hesperetin (Figure S2(b)). As the sugar moiety is hydrophilic (“water-loving”), it increases the water solubility of the molecule. The molecular structure of sugar contains OH groups, which participate in intermolecular hydrogen bonds with water molecules, resulting in the formation of hydrogen-bonded networks between water and the compounds. There are rhamnose and glucose moieties in NE, a glucose moiety in HMG, and no sugar moiety in hesperetin. The number of hydroxyl groups in NE is greater than that in HMG. Hesperetin has no sugar. Therefore, the water solubility of NE is the highest, and hesperetin is insoluble in water.

A comparison of the molecular structures of NA, prunin, and naringenin revealed that NA had the most sugar hydroxyl groups and that no sugar moiety was present in naringenin. Therefore, the water solubility sequence from high to low was inferred to be NA [17], prunin (Figure 4(b)), and naringenin (Figure 7(b)) based on the structure–solubility relationships of NE, HMG, and hesperetin. However, the dissolution curves of NA, prunin, and naringenin revealed that the compound with the highest water solubility was prunin but not NA, which conflicts with the water solubility theory. The water solubility theory must be modified to accommodate these conflicts.

In this study we prefer that the water solubility of organic compounds be determined by their aggregator structure, which is formed in water, rather than their single-compound structure [35,36]. The aggregator structure formed by the same compound changes when the compound exists in different solvents based on the intermolecular forces between the compound and the solvent. Moreover, in the same solvent, different compounds form different aggregators. The aggregator structure determines physical properties such as water solubility. To tentatively account for the unusual difference in water solubility between prunin and NA, we hypothesize that prunin may be capable of forming a relatively stable and soluble aggregator under our proposed model, whereas NA might lack this property.

As per our hypothetical model, prunin may form a hollow rod-shaped aggregator (Figure 10K) with surface-exposed sugars, contributing to solubility. NA, however, likely cannot form similar homogeneous hexamers. Its rhamnose moiety is oriented nearly vertically to glucose (Figure 10N). While this may slightly increase single-molecule solubility, during aggregation, the vertical rhamnose could sterically hinder NA approach, inhibiting inter-chain H-bond formation (Figure 10O) and preventing stable aggregator assembly, plausibly explaining NA’s lower solubility versus prunin.

The interpretation of the unexpected solubility trend via a putative ‘soluble aggregate’ or ‘hexamer’ model for prunin, while consistent with the observed dissolution profiles, remains a hypothesis. This model was proposed to reconcile the data with the initial hydroxyl-count-based prediction but requires direct experimental confirmation. It is highlighted here as one of the more speculative aspects of this work, pointing to a clear need for future investigation.

## 4. Conclusions

In this study, we assessed the impact of the sugar moieties present in NA and NE on the solubility of NA-NE c-ASD. The solubility profiles of NA with hesperetin-7-O-glucoside and hesperetin and of NE with naringenin-7-O-glucoside and naringenin was assessed by dissolution determination, and analyzed by PXRD. The results indicated that the rhamnose moiety and the glucose moiety on the NE sugar chain and the glucose moiety on the NA sugar chain are necessary for the formation of NE- NA c-ASD. A lack of sugar moiety results in an unstable aggregator structure and leads to a decrease in the concentration or a low water concentration in the water solution. The rhamnose moiety on the sugar chain of NA is removable. A stable soluble aggregator could also be constructed without it, but the soluble aggregator formation ability is weakened.

The insights into the role of sugar moieties in NA-NE c-ASDs provided by this study may inform the rational selection of co-formers for structurally similar flavanone glycosides. Furthermore, they suggest a hypothesis that targeted modification of c-ASD structures based on sugar moiety engineering could be a viable strategy to improve solubility or discover new aggregator entities, which warrants future validation on a broader range of structurally distinct glycosides.

The proposed optimal aggregate molar ratios, such as 1:2 for NE and prunin deduced from dissolution profiles at a fixed 1:1 weight ratio, require confirmation via ratio-scan experimental designs in future work; such validation would substantiate the stoichiometric interpretations and valuably extend the findings of this article. Moreover, further investigation into the structure–solubility relationship of sugar substituents—including those glycosylated at alternative aglycone hydroxyls or comprising longer carbohydrate chains—will advance the comprehension of how the sugar chain influences the formation mechanism of the NA-NE c-ASD.

## Figures and Tables

**Table 1:** Summary of the binary sample information.

Binary Sample	Weight Ratio	Molar Ratio	Removed Sugar Moiety
NE-prunin	1:1	0.71:1	the rhamnose moiety on NA
NE-naringenin	1:1	0.45:1	the glucose and rhamnose moieties on NA
HMG	1:1	0.80:1	the rhamnose moiety on NE
NA-hesperetin	1:1	0.52:1	the glucose and rhamnose moieties on NE

## Data Availability

The original contributions presented in this study are included in the article. Further inquiries can be directed to the corresponding authors.

## Supplementary Materials

The following supporting information can be downloaded at: https://www.mdpi.com/article/10.3390/pharmaceutics18081041/s1, Figure S1: The proposed aggregator model of NE-NA c-ASD (A) and NE-prunin c-ASD (B). Figure S2: Hesperetin dissolution profiles of the NA-hesperetin sample (a) and the single hesperetin sample (b).
