# Supplementary material for: The Impact of Sugar Moieties on the Solubility of Naringin-Neohesperidin Co-Amorphous Solid Dispersions
Source: Pharmaceutics. 2026 Aug 21;18(8):1041. doi: 10.3390/pharmaceutics18081041 (PMC13516785; doi:10.3390/pharmaceutics18081041)

Supplementary Materials

The Impact of Sugar Moieties on the Solubility of Naringin-Neohesperidin Co-Amorphous Solid Dispersions

Hua Jiang, Zhong-Kang Yang, Jun Li * and Yu-Pin Wang

College of Chemistry and Chemical Engineering, Henan University of Science and Technology, Luoyang 471023, China; jianghua@haust.edu.cn (H.J.); 240320251467@stu.haust.edu.cn (Z.-K.Y.); 240320251504@stu.haust.edu.cn (Y.-P.W.)

* Correspondence: lijun_pharm@haust.edu.cn

Figure Captions

Fig. S1. The proposed aggregator model of NE-NA c-ASD (A) and NE-prunin c-ASD (B).

Fig. S2. Hesperetin dissolution profiles of the NA-hesperetin sample (a) and the single hesperetin sample (b).

Fig. S1. The proposed aggregator model of NE-NA c-ASD (A) and NE-prunin c-ASD (B).

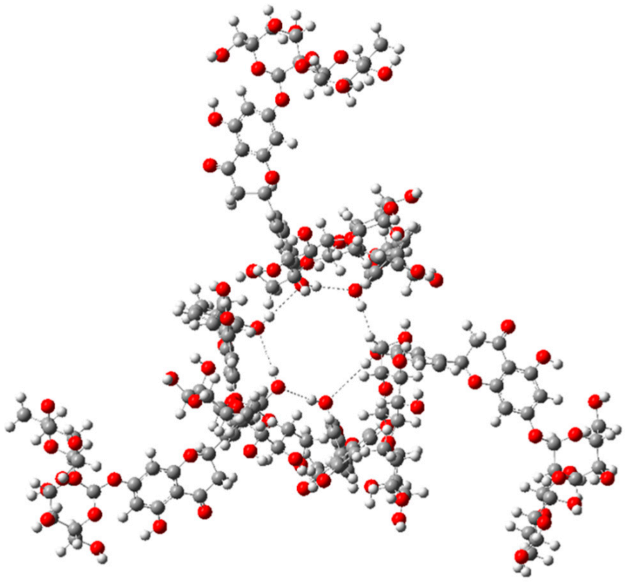

A

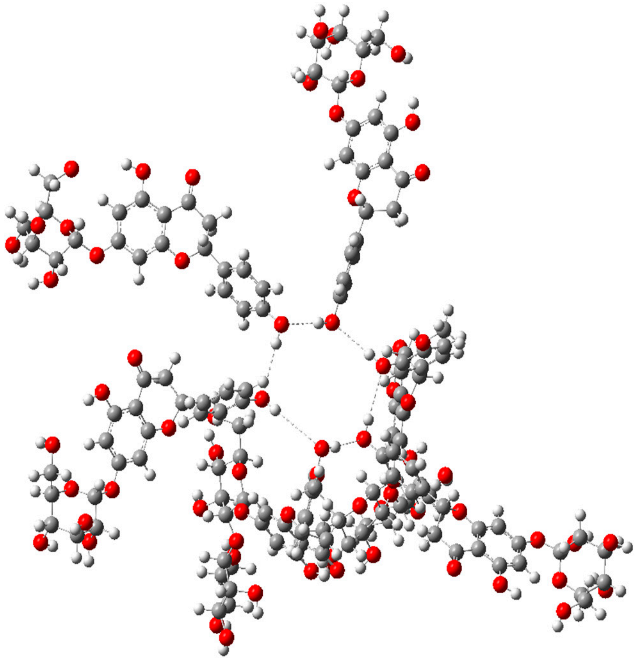

B

Fig. S2. Hesperetin dissolution profiles of the NA-hesperetin sample (a) and the single hesperetin sample (b).

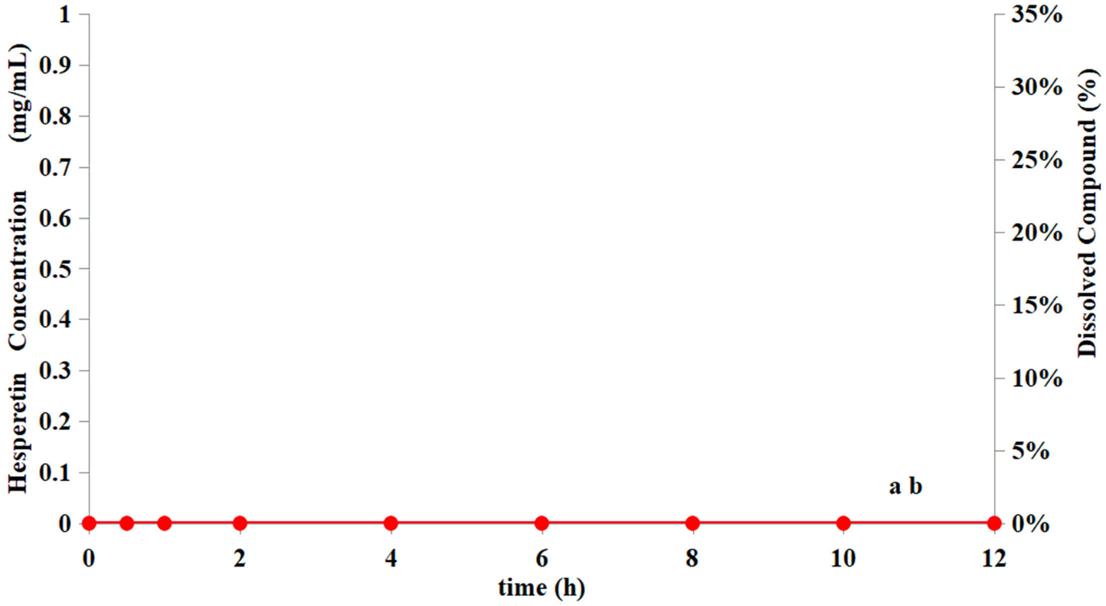

Supplement: Supplementary file 1 [file pharmaceutics-18-01041-s001.zip › pharmaceutics-4405583-supplementary.pdf]
